# Successful endoscopic diagnosis of angiosarcoma of the small intestine: A case report

**DOI:** 10.1002/deo2.24

**Published:** 2021-08-22

**Authors:** Koichi Tamura, Kenji Matsuda, Daisaku Ito, Toshihiro Sakanaka, Masato Tamiya, Takahiko Hyo, Masayuki Kitano, Hiroki Yamaue

**Affiliations:** ^1^ Second Department of Surgery School of Medicine Wakayama Medical University Wakayama Japan; ^2^ Second Department of Internal Medicine School of Medicine Wakayama Medical University Wakayama Japan

**Keywords:** angiosarcoma, biopsy, case report, obscure gastrointestinal bleeding, small intestine

## Abstract

A 51‐year‐old man had hematochezia, anemia, and an intraabdominal mass. Gastroscopy and colonoscopy showed no significant lesions with intraluminal bleeding, while radiological examinations showed bulky swelling of the lymph nodes around the abdominal aorta and a tumor in the left ischial bone. Small intestine endoscopy detected a dark purpuric protruding tumor of the jejunum and its biopsy specimen brought a definitive diagnosis of primary jejunal epithelioid angiosarcoma from positive staining of AE1/AE3, CD31, and erythroblast transformation specific related gene in immunohistochemical studies. The patient underwent surgical resection with adjuvant chemotherapy but died of progression of metastases 7 months after the diagnosis. Epithelioid angiosarcoma of the gastrointestinal tract is an extremely rare malignancy with poor prognosis and it is challenging to distinguish from undifferentiated carcinoma or melanoma. Immunohistochemistry is necessary for a definitive diagnosis. Sufficient biopsy specimen may aid a prompt diagnosis of this disease of the small intestine, which may present as obscure gastrointestinal bleeding.

## INTRODUCTION

We present a case of successful diagnosis of small intestinal angiosarcoma settled by endoscopic biopsy of the jejunum. Angiosarcoma is a rare malignant entity, accounting for just 1–2% of all types of sarcomas; it generally occurs in the skin and the subcutaneous tissue.^1^, ^2^ Primary angiosarcoma originating from the gastrointestinal tract is particularly rare, especially in the small intestine. Symptoms of small intestinal angiosarcoma are various and unstable, resulting in difficulty in making a quick definitive diagnosis, and its broad etiology can remain unknown for a long time. Most patients show poor clinical courses due to the progressive nature of metastases and scarcity of effective treatments after confirmed diagnosis. CASE REPORT

**FIGURE 1:**
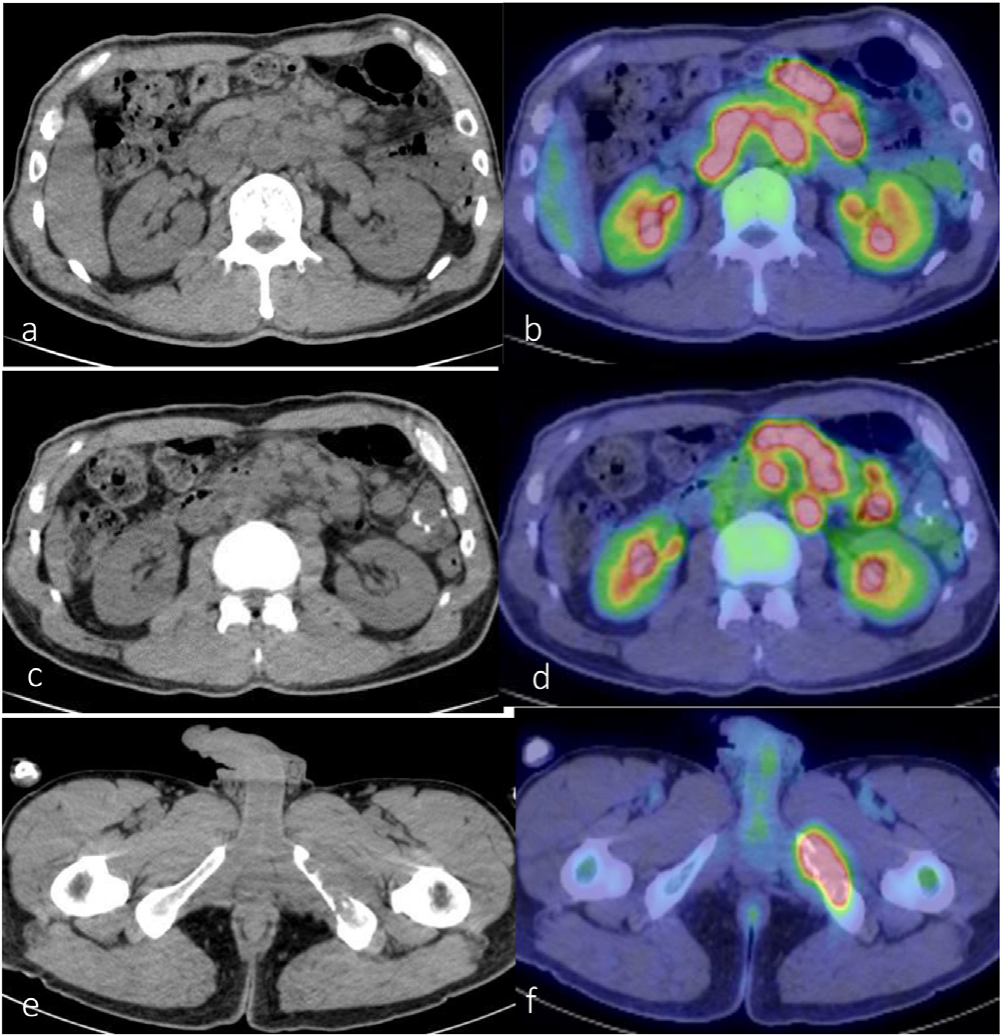
At the initial visit, computed tomography (CT) showed lymph nodes were swelled in heaps around the superior mesenteric artery and the abdominal aorta (a, c). Positron emission tomography / CT showed uptake of F‐18 fluorodeoxyglucose in these lymph nodes (b, d) and in the left ischial bone (e, f).

**FIGURE 2:**
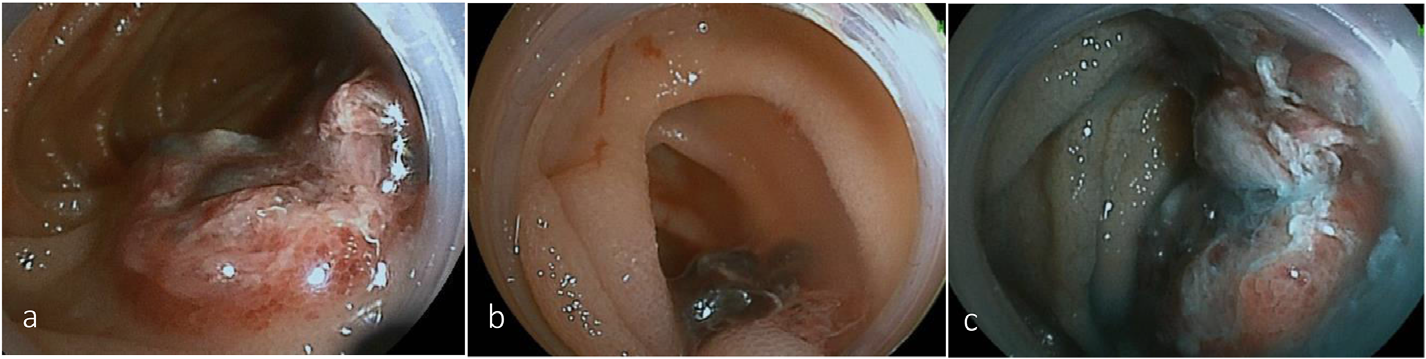
Small intestine endoscopy showed a gently protruded lesion composed of coarse nodules in the jejunum. This tumor involved a central foveation with dark purplish surface (a). The distal base of this lesion turned blackish brown (b). Chromoendoscopic imaging showed a flare mucosa with distinct foveation and mucus (c).

**FIGURE 3:**
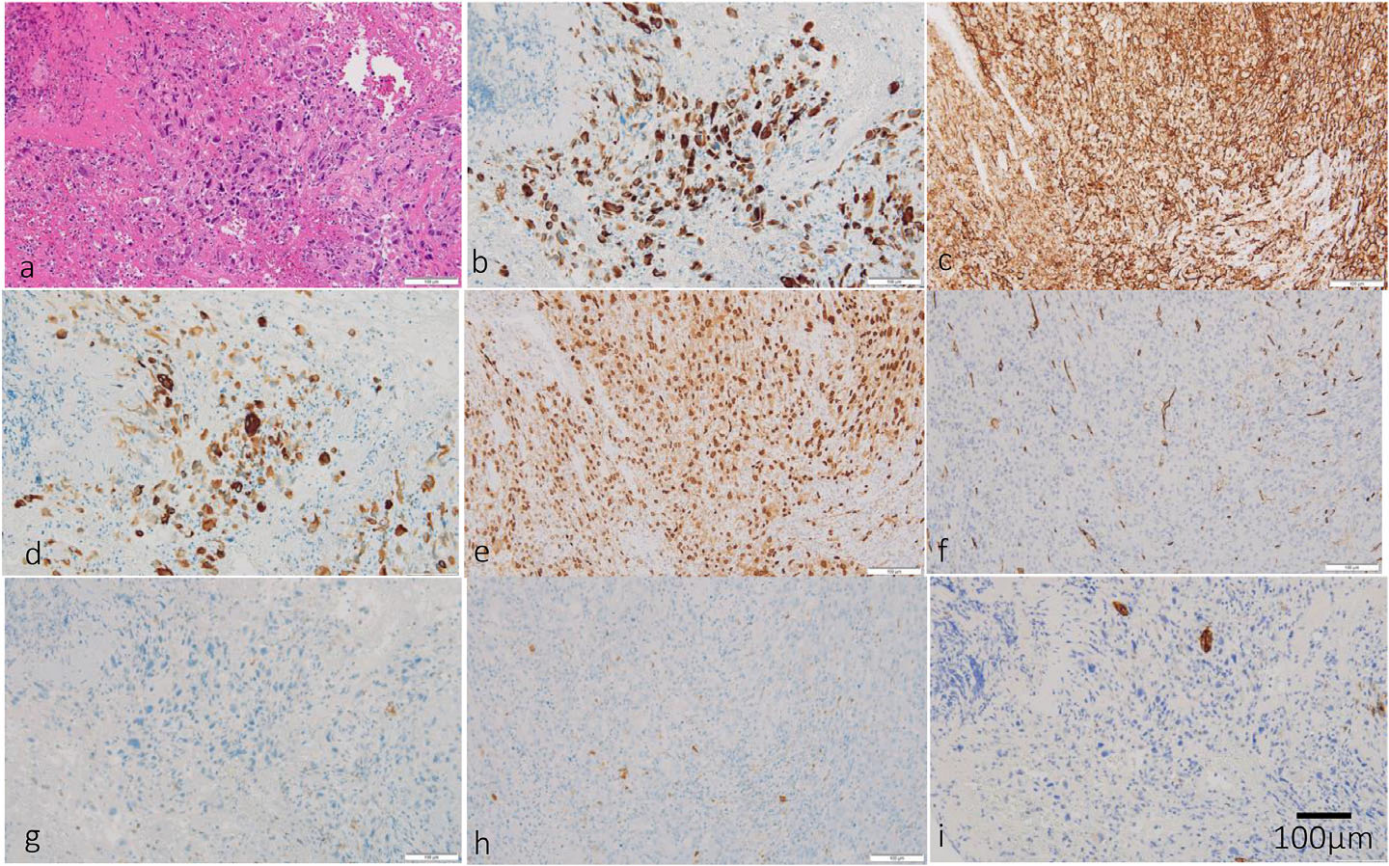
Histopathological findings of the biopsy specimen showed poorly differentiated carcinoma with pleomorphic change in hematoxylin and eosin staining × 200 (a). Immunohistochemical studies showed positive staining for AE1/AE3 (b), CD31 (c), CAM5.2 (d), and erythroblast transformation‐specific related gene (e) while showing negative staining for CD34 (f), CD20 (g), S‐100 (h), and epithelial membrane antigen (i), respectively × 200.

A 51‐year‐old man visited a clinic with tarry stool, loss of body weight, and severe anemia for a month. The patient was a government official and not an occupationally exposed person. Ultrasound scan showed bulky swelling of the lymph nodes around the superior mesenteric artery (SMA). He was presented to our hospital with a temporal diagnosis of malignant lymphoma for a detailed treatment. He was febrile and had fatigue, and laboratory tests showed anemia (hemoglobin: 6.2 g/dl, male normal range: 13.7–16.8 g/dl) and hypoalbuminemia (serum albumin: 2.6 g/dl, normal range: 4.1–5.1 g/dl). Other significant laboratory data were serum iron level 10 (normal range: 40–188 μg/dl) and serum soluble interleukin 2 receptor (sIL2R) level 775 (normal range: 127–582 U/ml). Computed tomography (CT) showed enlarged lymph nodes around the SMA to the abdominal aorta and segmental wall thickness of the jejunum (Figure 1a, c). F‐18 fluorodeoxyglucose positron emission tomography/CT showed abnormal uptakes in these lesions (Figure 1b, d) and in the left ischial bone (Figure 1e, f). Gastroscopy and colonoscopy detected no bleeding points. Consecutively, peroral small intestine endoscopy was carried out by board certificated endoscopists for fear of continuous jejunal bleeding, confirming that a dark purpuric protruding lesion with a centrally foveation was located 50 cm away from the Treitz ligament (Figure 2a). The surface of distal base turned blackish brown, which was not active bleeding but a suspected cause of bleeding (Figure 2b). Chromoendoscopic imaging showed a flare mucosa with distinct foveation and mucus (Figure 2c). These endoscopic appearances suggested intestinal melanoma, neuroendocrine tumor, metastatic carcinoma and angiosarcoma for differential diagnoses. We appropriately gained sufficient biopsy specimen and histopathological findings showed that epithelioid carcinoma with poorly differentiated and pleomorphic features in hematoxylin and eosin staining (Figure 3a). Immunohistochemical staining indicated that the tumor cells were positive for AE1/AE3, CD31, CAM5.2, and erythroblast transformation‐specific related gene (ERG), and negative for CD34, CD20, S‐100, and epithelial membrane antigen (EMA; Figures 3b–i). These results brought a compatible diagnosis with epithelioid angiosarcoma with multiple metastases. It took us 2 weeks to obtain this diagnosis after the patient's first visit. Additional capsule endoscopy was therefore postponed due to acquisition of this definitive diagnosis and apprehension of jejunal obstruction suggested by CT images.

**FIGURE 4:**
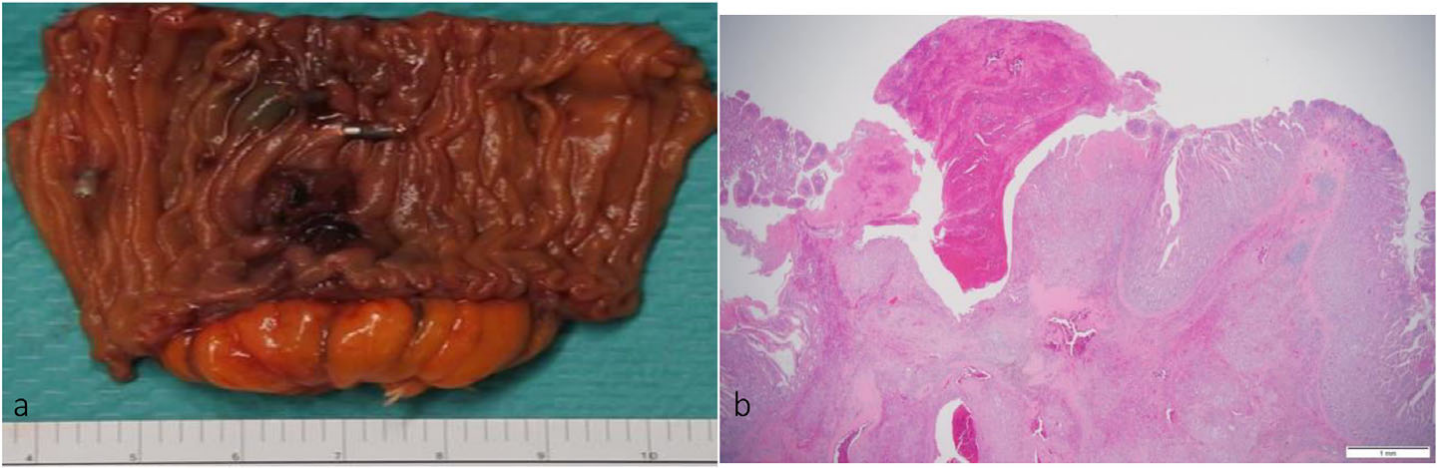
Resected specimen of the jejunum showed the tumor had infiltrated into the jejunal muscularis propia (a) gross appearance, (b) pathology image with a loupe in hematoxylin and eosin staining.

We performed laparoscopic partial resection of the jejunum as the patient's recovery from anemia was difficult, despite repeated blood transfusion. A protruding tumor of 10 × 12 mm in size was found in resected tissue of the jejunum (Figure 4a) and it had infiltrated into the muscularis propia (Figure 4b). The patient was recovering well in postoperative course and he was discharged 11 days after surgery. Histopathological features of surgical specimen supported the preoperative diagnosis identified by endoscopic biopsy.

Postoperatively, he received weekly administration of paclitaxel as a first line chemotherapy. His therapeutic regimen was then changed to doxorubicin because of allergic reaction. Rapid disease progression with cancer pain and cancerous cachexia resulted in his death 7 months after the diagnosis.

## DISCUSSION

Angiosarcoma is a rare malignancy of endothelial origin, which ranges from well‐differentiated tumor to high‐grade spindle cell type and a unique morphologic subtype of angiosarcoma that shows a predominantly epithelioid appearance with sheet‐like growth. This atypical epithelioid subtype has been defined as epithelioid angiosarcoma,^3^ originally described by Weiss et al.^4^ Its rapidly progressive growth following various metastases in nature creates difficulty in speedy diagnosis and in decision‐making of treatment strategies, leading to extremely poor prognosis.^5^ More than half of patients die of disease progression within 2 years of the diagnosis and most patients with metastatic lesions die within months.^3^, ^6^

The etiology of angiosarcoma is still unclear, although several risk factors (e.g., irradiation, exposure to vinyl chloride, thorotrast, and chronic lymphedema) relate to its pathogenesis.^2^ This patient had no compatible risk factors in his past history.

Definitive diagnosis of epithelioid angiosarcoma of gastrointestinal tract is quite difficult because of its various symptoms, insufficient specimen sampling and histopathological similarity of poorly differentiated carcinoma.^7^ Malignant lymphoma, intestinal melanoma, neuroendocrine carcinoma, metastatic carcinoma, and angiosarcoma were considered as our differential diagnoses from diagnostic imaging. Immunohistochemical examinations are indispensable for obtaining confirmed diagnosis. CD31expression has been reported to be a more specific marker of endothelial differentiation than CD34, and ERG has high sensitivity for detecting vascular differentiation, so CD31 and ERG staining may be suitable for the diagnosis of epithelioid angiosarcoma.^2^, ^8^, ^9^ Negative S‐100 protein staining suggests tumor cells have no neuroendocrine differentiation and no melanocytic proliferations.^3^ Negative EMA staining could potentially help a differential diagnosis from proximal‐type epithelioid sarcoma and mesothelioma.^2^, ^3^ Factor VIII related antigen is considered to be a useful marker of angiosarcoma,^2^ but was not performed in this case.

Treatment modalities for gastrointestinal epithelioid angiosarcoma include surgical resection, radiation therapy, and chemotherapy, which can be performed alone or in combination depending on the progression of each case.^10^ Most patients undergo surgical resection for definitive diagnosis and relief from clinical symptoms (e.g., gastrointestinal bleeding, bowel obstruction, and cancer pain). The median survival time in patients who undergo surgical resection with adjuvant chemotherapy is longer than that of surgery only.^10^ Our patient received adjuvant chemotherapy, but longer survival could not be obtained because of adverse events and drastic disease progression.

In conclusion, our patient with jejunal epithelioid angiosarcoma may be the first case to be successfully diagnosed by biopsy specimen from small intestine endoscopy. Gastroenterologists should consider this rare disease as a differential diagnosis for obscure gastrointestinal bleeding. We recommend immunohistochemical staining of CD34 and ERG positively and S‐100 and EMA negatively.

## CONFLICT OF INTEREST

The authors declare that there is no conflict of interest.

## ETHICS STATEMENT

The authors report the details of the patient's case in accordance with the ethical standards of the Declaration of Helsinki.

## FUNDING INFORMATION

None.
